# m^6^A methylation-mediated regulation of LncRNA MEG3 suppresses ovarian cancer progression through miR-885-5p and the VASH1 pathway

**DOI:** 10.1186/s12967-024-04929-x

**Published:** 2024-01-29

**Authors:** Yan Li, Shenghan Lou, Jian Zhang, Shilu Zhao, Ge Lou

**Affiliations:** 1 Department of Obstetrics and Gynecology, First Affiliated Hospital of Harbin Medical University, Harbin, 150007 Heilongjiang China; 2 Department of Gynecology, Harbin Medical University Cancer Hospital, 150 HaPing Road, Nangang District, Harbin, 150081 Heilongjiang China

**Keywords:** MEG3, m^6^A methylation, VASH1, Ovarian cancer

## Abstract

**Background:**

Ovarian cancer poses a serious threat to women's health. Due to the difficulty of early detection, most patients are diagnosed with advanced-stage disease or peritoneal metastasis. We found that LncRNA MEG3 is a novel tumor suppressor, but its role in tumor occurrence and development is still unclear.

**Methods:**

We investigated the expression level of MEG3 in pan-cancer through bioinformatics analysis, especially in gynecological tumors. Function assays were used to detect the effect of MEG3 on the malignant phenotype of ovarian cancer. RIP, RNA pull-down, MeRIP-qPCR, actinomycin D test were carried out to explore the m^6^A methylation-mediated regulation on MEG3. Luciferase reporter gene assay, PCR and Western blot were implemented to reveal the potential mechanism of MEG3. We further confirmed the influence of MEG3 on tumor growth in vivo by orthotopic xenograft models and IHC assay.

**Results:**

In this study, we discovered that MEG3 was downregulated in various cancers, with the most apparent downregulation in ovarian cancer. MEG3 inhibited the proliferation, migration, and invasion of ovarian cancer cells. Overexpression of MEG3 suppressed the degradation of VASH1 by negatively regulating miR-885-5p, inhibiting the ovarian cancer malignant phenotype. Furthermore, we demonstrated that MEG3 was regulated at the posttranscriptional level. YTHDF2 facilitated MEG3 decay by recognizing METTL3‑mediated m^6^A modification. Compared with those injected with vector control cells, mice injected with MEG3 knockdown cells showed larger tumor volumes and faster growth rates.

**Conclusion:**

We demonstrated that MEG3 is influenced by METTL3/YTHDF2 methylation and restrains ovarian cancer proliferation and metastasis by binding miR-885-5p to increase VASH1 expression. MEG3 is expected to become a therapeutic target for ovarian cancer.

**Supplementary Information:**

The online version contains supplementary material available at 10.1186/s12967-024-04929-x.

## Introduction

Epithelial ovarian cancer causes over 180,000 deaths worldwide each year, and 60% of cases are high-grade serous ovarian cancer (HGSOC) [1]. Ovarian cancer is characterized by a dormant onset, the absence of obvious symptoms in the early stages, and a lack of early diagnostic biomarkers; approximately 75% of patients have developed advanced disease by the time of diagnosis [2]. Although cytoreductive surgery accompanied by postoperative platinum-based chemotherapy has become a standardized treatment plan and radiotherapy, targeted therapy, and immunotherapy have enriched patient choices, the mortality rate of HGSOC has remained almost unchanged in recent decades, with a 5-year survival rate of approximately 30% [1]. Therefore, it is important to explore suitable biomarkers and their mechanisms in ovarian cancer.

In recent decades, long noncoding RNA (lncRNA) has evolved from being considered "transcription noise" to being considered a key regulatory factor in various cellular processes. LncRNAs represent a large and diverse set of ncRNAs with a length of over 200 nucleotides that can interact with RNA, DNA, and proteins, allowing them to regulate transcription and posttranscriptional processes through various mechanisms. LncRNAs are involved in various cellular processes from normal development to the occurrence of diseases, such as cancer [3, 4]. A growing amount of evidence suggests that lncRNAs can regulate the stability of messenger RNAs (mRNAs) in tumor tissue by acting as competitive endogenous RNAs (ceRNAs) or molecular sponges for microRNAs (miRNAs) [5–7]. LncRNA maternal expression gene 3 (MEG3) is an imprinted gene located on human chromosome 14q32.3 within DLK1-MEG3 locus that encodes a lncRNA-MEG3 RNA [8]. It contains 10 exons encoding approximately 1.6 kb of non-coding RNA [9]. The promoter of MEG3 includes TATA- and CCAAT box, and the RNA transcribed from this gene by RNA polymerase II is polyadenylation at the 3 'end [10, 11]. LncRNA MEG3 locates in the nucleus and cytoplasm [12]. Gene expression in the DLK1-MEG3 region is controlled by two differentially methylated regions (DMR) consisting of multiple methylated CpG sites: the intergenic DMR (IG-DMR) is located approximately 13 kb upstream of the MEG3 transcriptional initiation site, and the post fertilization-derived secondary (MEG3-DMR) overlapped with the upstream 1.5 kb promoter [13]. It has been found that MEG3 is downregulated in many kinds of tumors, including oral squamous cell carcinoma [14], head and neck squamous cell carcinoma [15], lung cancer [16], esophageal squamous cell carcinoma [17], colorectal cancer [18], renal cell carcinoma [19], bladder cancer [20, 21], prostate cancer [22], cervical cancer [23, 24], leukemia [25], and meningioma [26]. The low expression or absence of MEG3 is associated with large tumor size, advanced FIGO stage, deep infiltration, early metastasis, and low survival rate. The abnormal expression of MEG3 can inhibit the proliferation, migration, and invasion of tumor cells and promote tumor cell apoptosis. Therefore, MEG3 is considered a potential tumor suppressor factor [27]. However, research on MEG3 in ovarian cancer is not yet abundant or fully investigated.

N6-methyladenosine (m^6^A) is the most abundant form of RNA modification in eukaryotic cells, regulating gene expression and cell fate. m^6^A is dynamically regulated by methyltransferases (“writers”), demethylases (“erasers”), and mA-binding proteins (“readers”). By binding different “readers”, m^6^A modification can affect the entire RNA life cycle, including alternative polyadenylation and pre-RNA processing, RNA export from the nucleus to the cytoplasm, translation, and decay [28, 29]. For example, YTH N6-methyladenosine RNA-binding protein 2 (YTHDF2) is a member of the m^6^A reader family, whose function is to recognize m^6^A modification sites on RNAs and promote their degradation [30, 31].

Here, we investigated the expression level of MEG3 in ovarian cancer and observed that increased MEG3 expression prolonged the overall survival (OS) of patients. We demonstrated that MEG3 could inhibit the proliferation and migration of ovarian cancer cells. Furthermore, from a mechanistic perspective, we found that MEG3 was regulated by METTL3-mediated YTHDF2 methylation, which promotes its degradation. More importantly, this study comprehensively verified the regulatory effect of the MEG3/hsa-miR-885-5p/VASH1 axis on the development of ovarian cancer. Finally, we also validated the inhibitory effect of MEG3 on ovarian cancer in a xenograft model. Overall, our research revealed the role of MEG3 in obstructing the progression of ovarian cancer from a new perspective, which may provide novel target insights for the future treatment of ovarian cancer.

## Materials and methods

### Databases

Gene Expression Profiling Interactive Analysis (GEPIA, http://gepia.cancer-pku.cn) is a web server for cancer and normal tissue (including TCGA normal and GTEx data) gene expression analysis and interaction analysis. It was used to explore common genes with differential expression in gynecological tumors. The Cancer Genome Atlas (TCGA, portal.gdc.cancer.gov/) was used to compare the differential expression of MEG3 across cancers. The TNM plot (https://tnmplot.com/analysis/) can analyze distinct gene expression in tumor, normal, and metastatic tissues. This database was used to compare the expression levels of MEG3 in pancancer tissues and normal tissues. The PrognoScan database (http://dna00.bio.kyutech.ac.jp/PrognoScan/index.html) contains a large array database of cancer samples with prognostic information. It was utilized to calculate the biological relationship between gene expression levels and clinical factors. ENCORI (The Encyclopedia of RNA Interactomes, http://starbase.sysu.edu.cn/index.php) is a comprehensive database specifically designed for RNA interactions. We exploited it to predict RNA binding sites. SRAMP (http://www.cuilab.cn/sramp) served as a useful tool to predict m^6^A modification sites on the RNA sequences of MEG3. Rm2target (http://rm2target.canceromics.org/#/home) is the first comprehensive database resource focusing on different WER targets modified by different RNAs.

### Cell lines and cell culture

All ovarian cancer cell lines (A2780 RRID: CVCL_0134, Caov-3 RRID: CVCL_0203, OVCAR-3 RRID: CVCL_0465, and SK-OV-3 RRID: CVCL_0532) and a normal epithelial cell line (IOSE80, RRID: CVCL_5546) were purchased from Procell Life Technology Co., Ltd. (Wuhan, China) with supporting STR cell line authentication. The cells were cultured in RPMI-1640 medium (Gibco Company, New York, USA) containing ten percent fetal bovine serum, 100 µg/ml streptomycin, and 100 IU/ml penicillin in a 37 °C incubator with 5% CO_2_.

### Quantitative real-time PCR

RNA was purified using the Total RNA Kit (Omega), and a total of 1000 μg RNA was reverse transcribed into cDNA using the RT‒PCR Transcriptor First Strand cDNA Synthesis Kit (Roche). qPCR was performed to detect the mRNA expression level using FastStart Universal SYBR Green Master Mix (ROX) (Roche) with the Applied Biosystems StepOnePlus™ Real-Time PCR system (Thermo Fisher Scientific, Inc., USA). Human β-actin and U6 were used as internal references to determine mRNA and microRNA expression. The expression levels were calculated using the 2-ΔΔCq method.

### Lentiviral vector infection and transient transfection

Lentiviruses expressing MEG3, scrambled shRNA, and empty vector were purchased from the General Biology Company. SKOV3 cells were selected to establish stable MEG3 overexpression cell lines. A2780 cells were used to establish MEG3 knockdown cell lines. A total of 5*10^4^ cells were plated into a 6-well plate and transfected with the indicated lentivirus using polybrene in accordance with the manufacturer’s instructions. Infected cells were selected using 2 μg/ml puromycin (MCE, USA) for ≥ 1 week, and the transfection efficiency was determined by qRT‒PCR and western blotting analysis. The sequences of METTL3, YTHDF2, hsa-miR-885-5p, and VASH1 were obtained from the National Center of Biotechnology Information. A2780 or SKOV3 cells were seeded into a 6-well plate. When a single layer formed, the culture medium was discarded. Next, the cells were transfected with the following plasmids: (1) blank, mimic-NC, hsa-miR-885-5p mimic, inhibitor-NC, hsa-miR-885-5p inhibitor; (2) blank, siRNA-NC, siRNA-METTL3, pcDNA-NC, and pcDNA-METTL3; (3) blank, siRNA-NC, siRNA-YTHDF2, pcDNA-NC, and pcDNA-YTHDF2; and (4) blank, siRNA-NC, siRNA-VASH1, pcDNA-NC, and pcDNA-VASH1. All plasmids used in the present study were constructed by General Biology Company (Anhui, China).

### DNA synthesis assay

The EdU Apollo 567 in vitro imaging kit (RiboBio, Guangzhou, China) was used according to the manufacturer’s protocol. EdU was added to each cell and incubated for 2 h. Cells were fixed with 4% paraformaldehyde for 30 min and permeabilized with 0.5% Triton-X 100 for 10 min. Cells were stained with Apollo 567 and Hoechst 33,342 for 30 min and scanned with a high-content imaging system (Cellomics ArrayScan VTI, Thermo Fisher Scientific, Carlsbad, CA, USA).

### Wound healing assay

Cells were seeded in 6-well plates and cultured until they covered the entire monolayer. A (yellow) pipette tip was used to draw a straight scratch to form a wound. Healing was observed under a microscope at 0, 12, 24, and 36 h, and a series of photographs were taken to document the process.

### Colony formation assay

The cells were seeded into a 6-well plate at a density of 500 cells/well. At 37 °C and 5% CO_2_, the cells were cultured for 2 weeks, and the solution was replaced with fresh RPMI-1640 every 2 days. The culture was terminated when visible colonies were observed. The cells were then washed with PBS and fixed with 4% paraformaldehyde for 15 min at room temperature. Finally, the cells were stained with 0.1% crystal violet (Solarbio, China) for 20 min, and the staining solution was removed by washing. The colonies were photographed using a digital camera.

### Proliferation assay

A total of 3*10^3^ cells were cultivated in a 96-well plate, and the culture solution was replaced with fresh RPMI-1640 every day. Cell Counting Kit-8 (BiYunTian, China) assays were used to test cell proliferation after 24, 48, and 72 h of incubation.

### Flow cytometry analysis of cell cycle and apoptosis

For the cell cycle analysis, 10^6^ cells were seeded into 6-well plates. After 48 h of transfection, the cells were collected and fixed in chilled 70% ethanol at − 20 °C overnight, followed by washing with phosphate-buffered saline. The fixed cells were stained with 50 mg/ml propidium iodide (PI) at room temperature for 20 min. For the apoptosis assay, the cells were stained with annexin V-FITC/PI (MultiSciences Biotech, Hangzhou, China). Flow cytometry analysis was performed using a FACSCalibur system (BD Biosciences, USA).

### Cell migration and invasion assay

Cell invasion ability and migration ability were measured using a Transwell chamber (8-μm pore size, Corning Costar, USA) precoated with or without Matrigel. A total of 2*10^4^ cells suspended in serum-free DMEM were seeded evenly into the upper chamber, and the lower chamber contained medium supplemented with 10% FBS. After 48 h of incubation, the filters were fixed in methanol and stained with 0.5% crystal violet. The cells on the upper side of the membrane were gently removed, and the cells that passed through the membrane were imaged and counted under the microscope.

### RNA stability assay

Cells were seeded in 6-well plates, and siRNAs were used to knock down or overexpress specific expression for 48 h. Then, the cells were treated with actinomycin D (CST, 5 μg/ml) for 0, 1, 2, 3, and 4 h. The target RNA expression level was measured by qRT‒PCR.

### Western blot analysis

Integral proteins were collected using RIPA buffer (BiYunTian, China). A bicinchoninic acid (BCA) kit (Biotech, China) was used to determine protein concentrations. A total of 20 μg of protein was separated using 12.5% SDS‒PAGE and transferred onto a PVDF membrane (Bio-Rad). After blocking with five percent skim milk for one hour at temperature, the membrane was subsequently incubated in a solution of diluted antibody.

### Luciferase reporter gene assay

The recombinant reporter gene plasmid MEG3-Wt or MEG3-Mut and hsa-miR-885-5p mimic were cotransfected into ovarian cancer cells. Similarly, VASH1-Wt or VASH1-Mut and hsa-miR-885-5p mimics were cotransfected into cells. After 48 h of plasmid cotransfection, the culture medium was discarded, and 100 μl of 1X PBS was used to wash the monolayer cells once. Then, 50 μl of 1X PLB was added to each well and shaken for 15 min at room temperature for lysis. Luciferase assay buffer II (100 μl) and cell lysis buffer (10 μl) were added to each well (Promega, America). After mixing them for 30 s, their firefly luciferase activity was plotted. Then, 100 μl of Stop&Glo buffer was added to immediately quench firefly luciferin and stimulate renilla luciferase.

### Subcellular fractionation

To determine the subcellular location of LncRNA MEG3, nuclear and cytoplasmic RNA were isolated using the E.Z.N.A.® Total RNA Kit (R6834, OMEGA) and Cytoplasmic/Nuclear RNA Purification Kit (Cat. 21000, NORGEN) according to the instructions. The expression was measured by qRT‒PCR.

### RNA immunoprecipitation (RIP) assays

RIP assays were performed using the RIP RNA Binding Protein Immunoprecipitation Kit (SaiCheng, KT102-01) according to the instructions. IP lysis buffer was used to lyse A2780 cells. Cell lysate combined with 5 μg magnetic beads of specific antibodies was incubated overnight at 4 °C. After washing, the lysates were digested with protease K, and the RNA that bound to the immunoprecipitated protein was purified.

### RNA pull-down

Using the T7 RiboMAXTM Express Large-Scale RNA Production System (P1320, Promega), abundant biotin-labeled MEG3 and antisense stranded RNA were transcribed in vitro. Biotinylated RNA was incubated with proteins extracted from A2780 cells and mixed with precleared streptavidin beads. According to the manufacturer’s protocol of the PierceTM Magnetic RNA Protein Pull-Down Kit (Cat. 20164, Thermo Fisher), beads were collected by centrifugation and washed with RNA washing buffer. RNA‒protein complexes were eluted, denatured, and processed by SDS/PAGE.

### MeRIP assays

MeRIP assays were performed following the manufacturer’s instructions (Magna MeRIP m6A Kit).

### Orthotopic xenograft models

The in vivo experimental procedures were approved by the Institutional Animal Care and Use Committee of the Center of Harbin Medical University and conformed to all regulatory standards. All animals were five-week-old female BALB/c nude mice purchased from Charles Rivers, Beijing, China. A total of 5*105 A2780 cells transfected with empty vector or MEG3 knockdown cells were suspended in PBS, mixed with Matrigel, and injected into the armpit of nude mice. Subsequently, we monitored the tumor volume with a Vernier caliper every 3 days for 4 weeks and used the formula (width 2 × length)/2 (mm^3^) for volume calculation. Mice were deeply anesthetized by intraperitoneal injection of sodium thiopental before tumour planting and euthanized at the end of the experiment.

### IHC assay

The tissue slices were dried at 70 ℃ for 3 h, followed by dewaxing and rehydration. Subsequently, the slices were rinsed with PBS. The washed slices were treated in the dark with 3% H2O2 for 15 min. After washing in distilled water, the slices were washed again with PBS. Antigen recovery was performed in citrate buffer (pH 6.0). Each slice was left overnight with rabbit anti-VASH1 polyclonal primary antibodies (Abcam, Cambridge, MA, USA) diluted at 1:100. The sections were washed three times with PBS and incubated with anti-rabbit secondary antibodies (1:200; Abcam, Cambridge, MA, USA) at room temperature for an additional 30 min. Each section was immersed in 500 μl of diaminobenzidine working solution and washed with PBS at room temperature for 10 min. Finally, the slides were restained with hematoxylin and fixed in Crystal Mount medium. The expression of VASH1 was independently analyzed and scored by two observers based on the intensity and distribution of positively stained tumor cells, which were divided by yellow particles in the cytoplasm. The staining index was evaluated as follows: > 70% of tumor nuclei with no detected staining, negative (−); ≥ 30% of tumor nuclei with weak staining, weakly positive ( +); and > 30% of tumor nuclei with strong staining (invisible nucleoli), strong positive (+ +).

### Statistical analysis

Each experiment was repeated independently at least thrice. All statistical analyses were performed with SPSS 22.0 and GraphPad Prism software. P values were calculated using the unpaired Student’s t test. A P value < 0.05 was considered to indicate statistical significance.

## Results

### MEG3 expression is decreased in gynecological malignant tumors and is associated with the prognosis of ovarian cancer patients

**Fig. 1:**
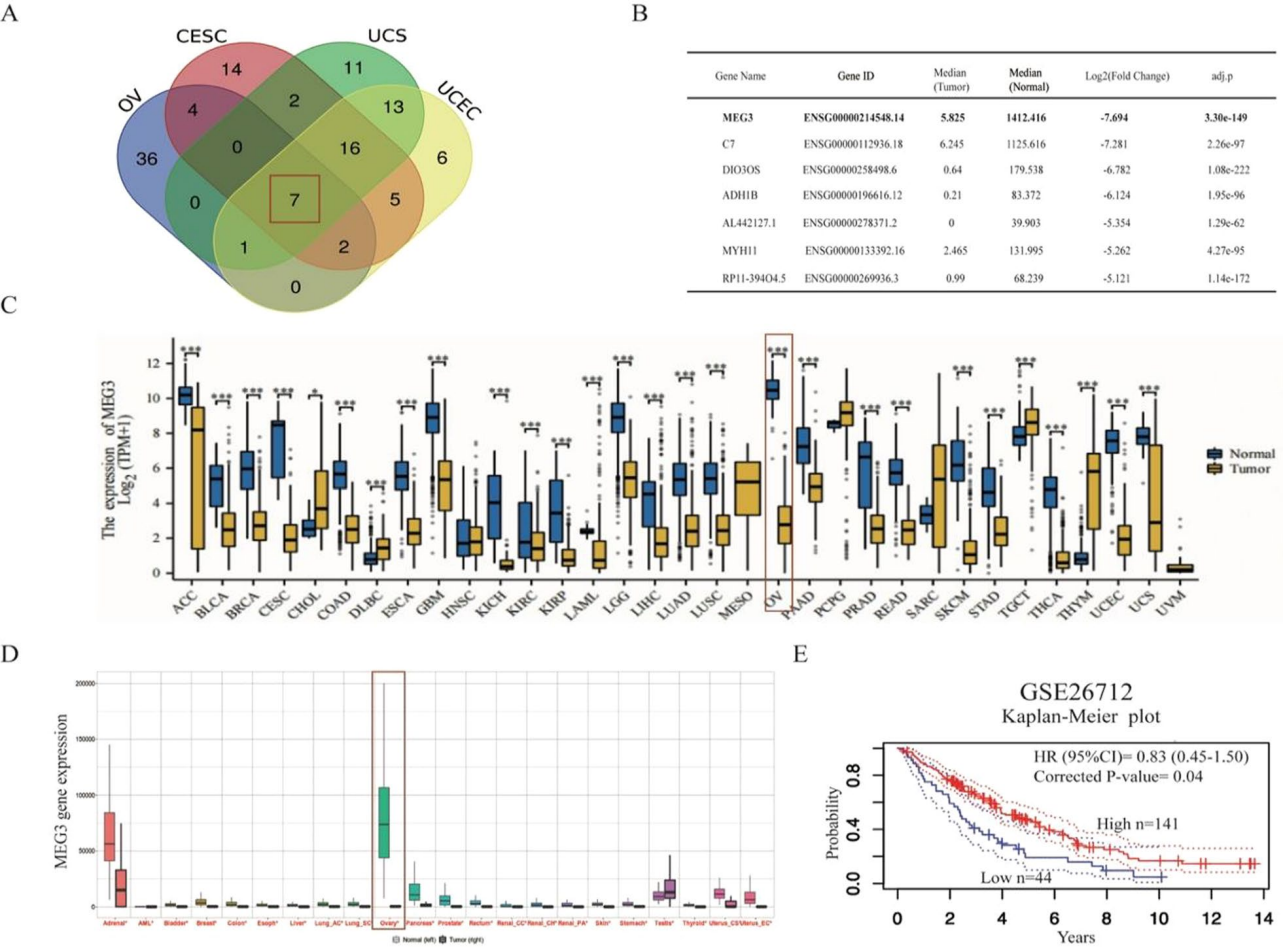
MEG3 expression is reduced in ovarian cancer, and this phenotype is associated with better prognosis. **A** The Venn diagram displays seven common genes that were downregulated in gynecological tumors. **B** Detailed information on seven commonly differentially expressed genes is listed. **C**, **D** In the TCGA and TNM plot databases, the expression level of MEG3 in pancancer and normal tissues was the most significantly different in ovarian cancer. **E** High expression of MEG3 was associated with better overall survival in ovarian cancer patients

We used the GEPIA database to explore the top 50 downregulated genes in gynecological malignancies, including ovarian cancer, cervical cancer, endometrial cancer, and uterine sarcoma. The name of these genes were listed in Additional file 1. Then, we intersected these genes and obtained seven simultaneously downregulated genes in gynecological malignant tumors (Fig. 1A). We sorted them based on the expression fold change in OV vs. normal ovary tissues and found that MEG3 ranked first (Fig. 1B). Subsequently, we found that MEG3 expression was decreased in pancancer tissues compared to normal tissues, and the fold change was highest in ovarian cancer (Fig. 1C). We further confirmed the above findings through the TNMplot database (Fig. 1D). The analysis results of OS were not statistically significant by using the TCGA database. By analyzing the GSE26712 dataset, we found that patients with high MEG3 expression had longer OS than those with low MEG3 expression (Fig. 1E).

### MEG3 inhibited the proliferation and migration of ovarian cancer cells

**Fig. 2:**
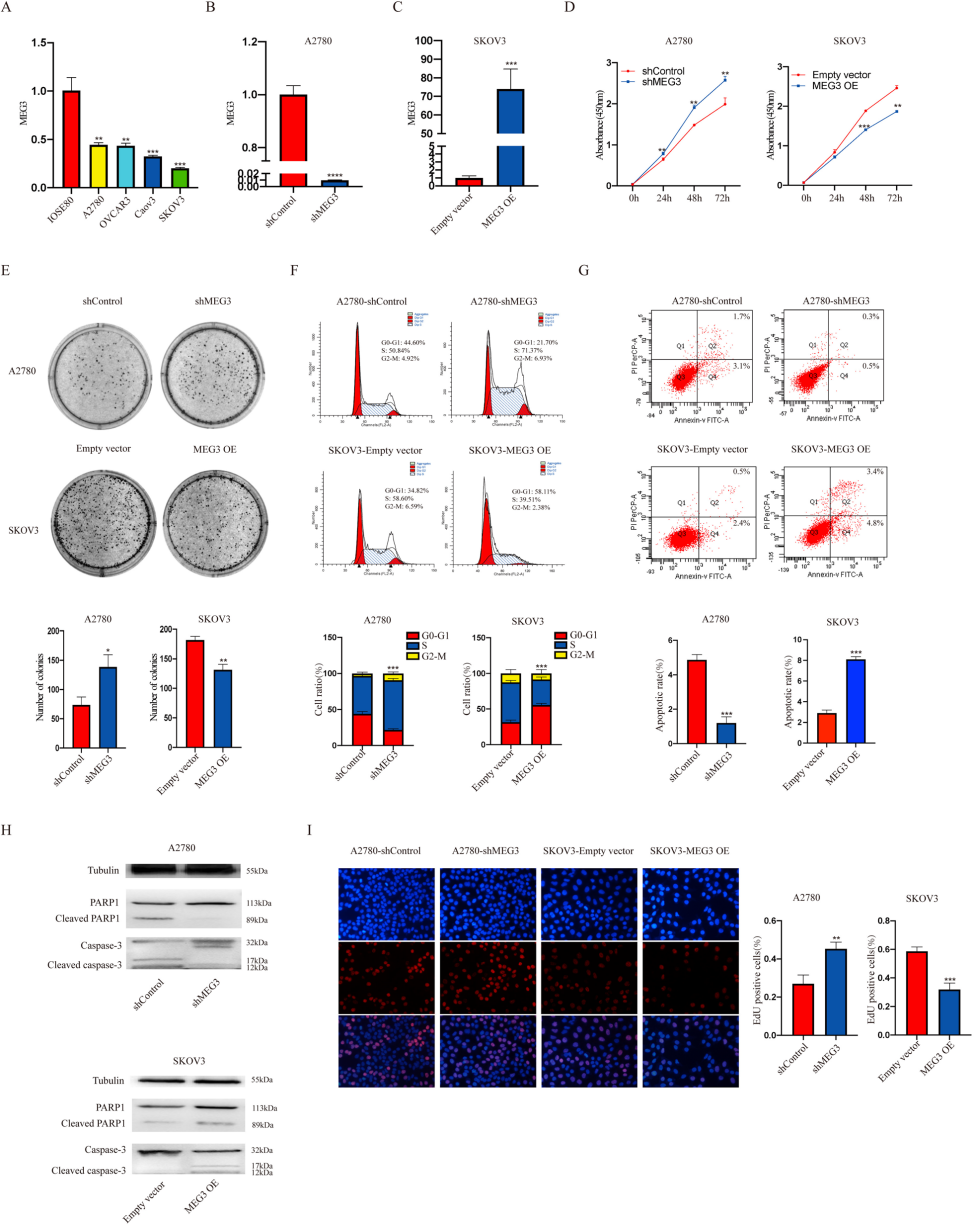
MEG3 can inhibit the proliferation of ovarian cancer cells. **A** Expression levels of MEG3 in normal ovarian epithelial cell lines and ovarian cancer cell lines. **B**, **C** qRT‒PCR was used to verify the construction of MEG3 knockdown (A2780) and overexpression (SKOV3) cell lines. **D** CCK-8 assay of NC, MEG3 knockdown, and MEG3 overexpression cells. **E** Colony formation assay of NC, MEG3 knockdown, and MEG3 overexpression cells. **F** Cell cycle analysis of NC, MEG3 knockdown, and MEG3 overexpression cells. **G** Apoptosis analysis of NC, MEG3 knockdown, and MEG3 overexpression cells. **H** Expression level of caspase-3, cleaved caspase-3, PARP1, and cleaved PARP1 in NC, MEG3 knockdown, and MEG3 overexpression cells. **I** DNA synthesis assay of NC, MEG3 knockdown, and MEG3 overexpression cells. *P < 0.05; **P < 0.01; ***P < 0.001; ****P < 0.0001

The expression levels of MEG3 in ovarian cancer cell lines (A2780, OVCAR3, Caov3, and SKOV3) and ovarian normal epithelial cells were verified by PCR. Compared with normal ovarian epithelial cells, MEG3 expression was generally reduced in ovarian cancer cells (Fig. 2A). To verify the effect of MEG3 on the biological phenotype of ovarian cancer cells, we established MEG3 overexpression and knockdown cell lines in SKOV3 and A2780 using lentivirus infection, respectively, and proved the transfection efficiency using qRT‒PCR (Fig. 2B, C). Then, we conducted a CCK-8 assay and found that MEG3 overexpression significantly reduced the proliferation of ovarian cancer cells (Fig. 2D). Next, the colony formation experiment results showed that knocking down MEG3 enhanced the cell colony formation ability, while overexpressing MEG3 slowed the cell cloning rate (Fig. 2E). In addition, the results of the cell cycle and apoptosis assays showed that knocking down MEG3 in A2780 cells led to an increase in cell proliferation and a decrease in the cell apoptosis rate. Overexpression of MEG3 in SKOV3 cells resulted in the opposite effect (Fig. 2F–H). The EdU experiment confirmed the inhibitory effect of MEG3 on DNA synthesis (Fig. 2I).

**Fig. 3:**
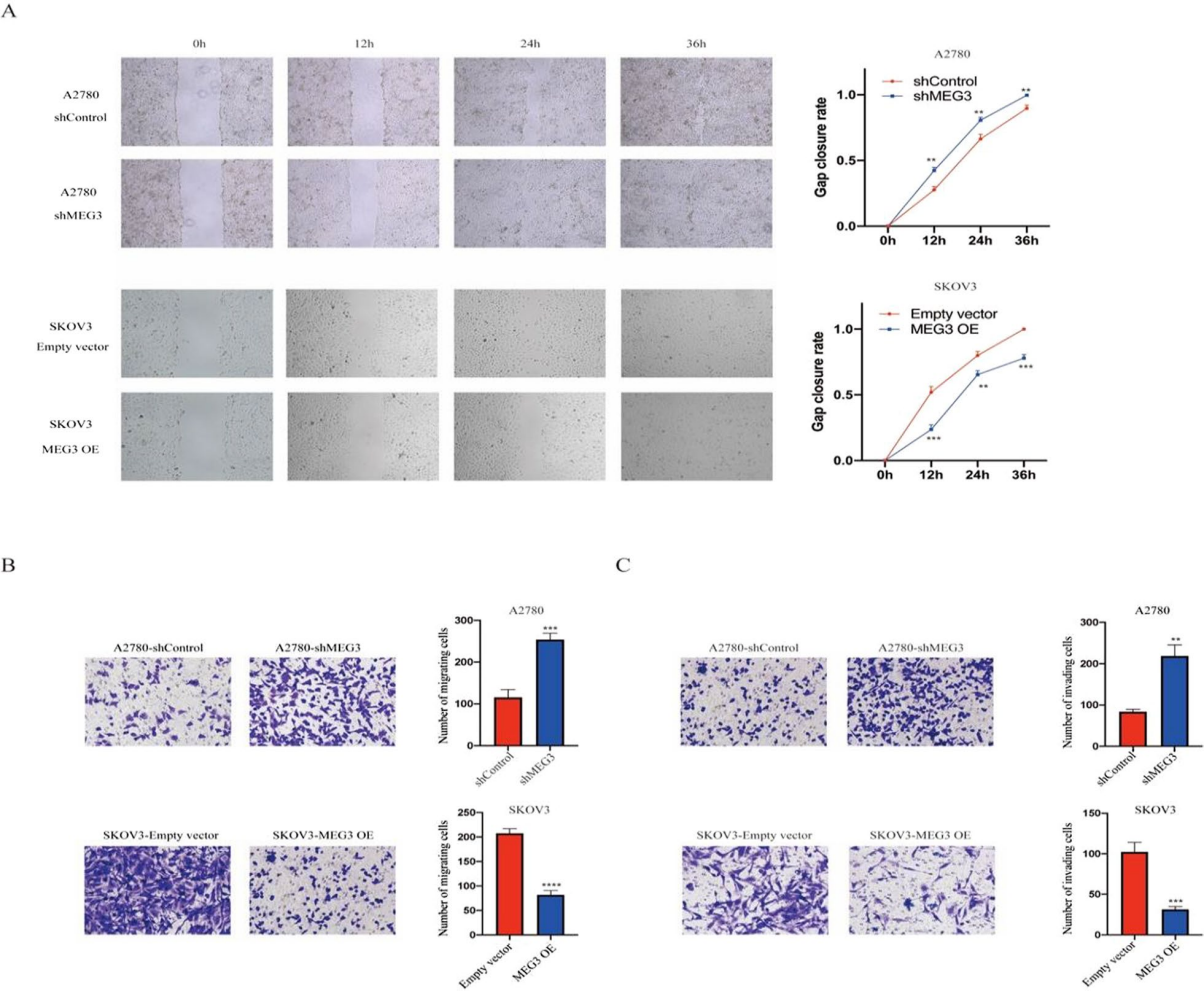
MEG3 can decrease the migration ability of ovarian cancer cells. **A** Scratch assay of NC, MEG3 knockdown, and MEG3 overexpression cells. **B** Migration assay of NC, MEG3 knockdown, and MEG3 overexpression cells. **C** Invasion assay of NC, MEG3 knockdown, and MEG3 overexpression cells. **P < 0.01; ***P < 0.001; ****P < 0.0001

Then, we performed wounding healing assays and found that knocking down MEG3 significantly reduced the gap closure rate of A2780 cells, while overexpression of MEG3 accelerated the wound closure rate of SKOV3 cells (Fig. 3A). Moreover, MEG3 knockdown markedly enhanced the migration and invasion of A2780 cells. In contrast, MEG3 overexpression obviously suppressed the migration and invasion of SKOV3 cells (Fig. 3B, C). Meanwhile, we also validated the impact of knocking down MEG3 on phenotype in SKOV3 cells, and the results were consistent with the above (Additional file 2). In summary, research results indicated that MEG3 inhibited ovarian cancer cell malignancy.

### YTHDF2 accelerated MEG3 decay via METTL3-mediated m^6^A modification

**Fig. 4:**
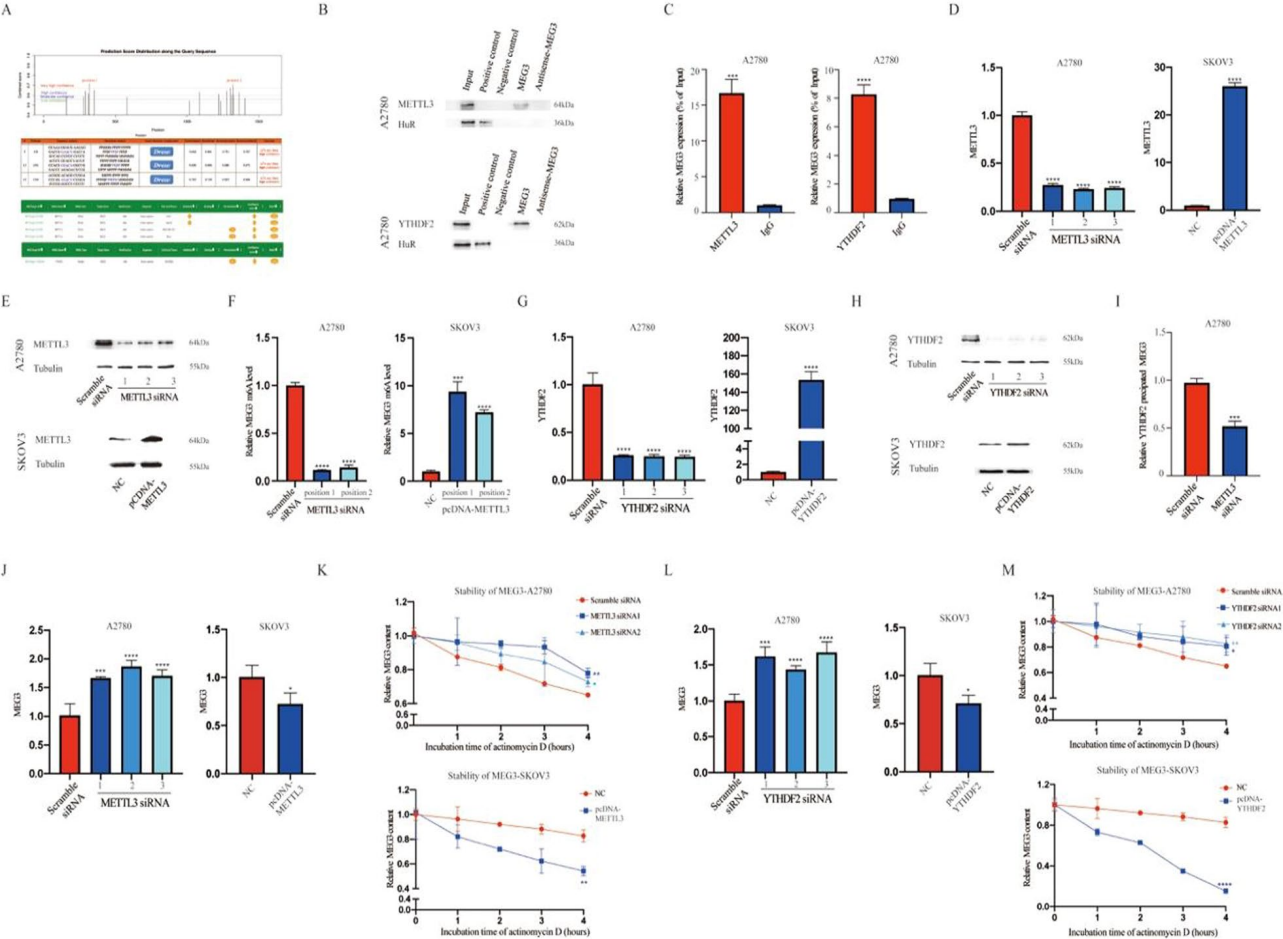
METTL3-mediated YTHDF2 methylation prevented MEG3 degradation. **A** Prediction of MEG3 methylation sites and binding proteins. **B** RNA pull-down showed the binding content of MEG3 with METTL3 and YTHDF2. **C** RIP assay showed the content of MEG3 immunoprecipitated by METTL3 and YTHDF2 antibodies. IgG antibodies were used as a negative control. **D**, **E**, **G**, **H** qRT‒PCR and western blotting validation of METTL3 and YTHDF2 transfection efficiency. **F** MeRIP-PCR showed METTL3 positively regulating MEG3 methylation levels. **I** RIP assay showed the content of MEG3 immunoprecipitated by YTHDF2 antibodies in A2780 cells with or without METTL3 siRNA. **J** The expression levels of MEG3 in A2780 cells with or without METTL3 siRNA and in SKOV3 cells with or without pcDNA-METTL3. **K** The stability of MEG3 in A2780 cells with or without METTL3 siRNA and in SKOV3 cells with or without pcDNA-METTL3. **L** The stability of MEG3 in A2780 cells with or without YTHDF2 siRNA and in SKOV3 cells with or without pcDNA-YTHDF2. **M** The stability of MEG3 in A2780 cells with or without YTHDF2 siRNA and in SKOV3 cells with or without pcDNA-YTHDF2. *P < 0.05; **P < 0.01; ***P < 0.001; ****P < 0.0001

To explore the influence of MEG3 on m^6^A methylation regulation, we first predicted methylation sites through the SRAMP website and obtained three sites with very high confidence. Next, the m^6^A methylation-related “writer” and “reader” proteins modifying MEG3 were predicted through the RM2Target website. METTL3 and YTHDF2 were highly credible, as they have been demonstrated to have binding regulatory effects with MEG3 in various cells (Fig. 4A). Next, an RNA pull-down assay indicated that METTL3 and YTHDF2 could directly interact with MEG3 in A2780 cells (Fig. 4B). Furthermore, RIP experiments demonstrated that compared to IgG, the RNA precipitated by METTL3 and YTHDF2 had higher levels of MEG3 expression (Fig. 4C). Next, we investigated the effect of METTL3 on MEG3 methylation level. We constructed METTL3 knockdown and overexpressing cell lines through plasmid transfection and verified them by qRT‒PCR and western blotting (Fig. 4D, E). The results of MeRIP-PCR showed that METTL3 positively regulated MEG3 methylation levels (Fig. 4F). Then, we constructed YTHDF2 knockdown overexpressing cell lines through plasmid transfection and verified them by qRT‒PCR and western blotting (Fig. 4G, H). The amount of MEG3 precipitated by the YTHDF2 antibody was also significantly decreased in METTL3-knockdown cells (Fig. 4I). Additionally, we explored the effect of METTL3 and YTHDF2 on MEG3 expression. We found that MEG3 expression increased after knocking down METTL3 in A2780 cells but decreased in SKOV3 cells overexpressing METTL3 (Fig. 4J). Similarly, MEG3 expression increased with YTHDF2 knockdown in A2780 cells and was reduced with YTHDF2 overexpression in SKOV3 cells (Fig. 4L). In addition, we validated the influence of METTL3 and YTHDF2 on the stability of MEG3. The stability of MEG3 was increased in both METTL3 knockdown cells and YTHDF2 knockdown cells but decreased in both METTL3- and YTHDF2-overexpressing cells (Fig. 4K, M).

### VASH1 expression was regulated by MEG3

**Fig. 5:**
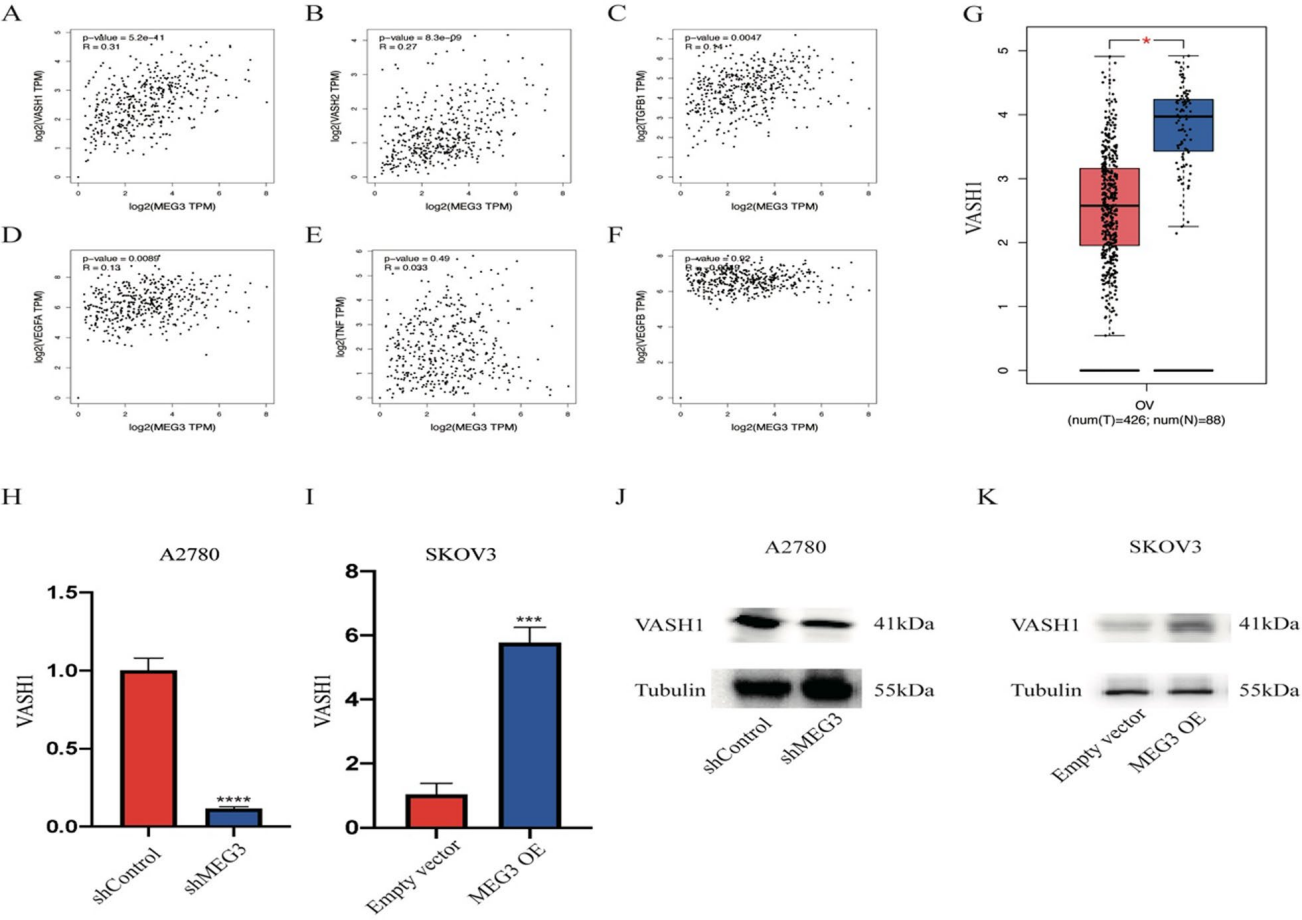
MEG3 positively regulates VASH1. Correlation of MEG3 expression with angiogenesis-related factors, including **A** VASH1, **B** VASH2, **C** TFGB1, **D** VEGFA, **E** TNF, and **F** VEGFB. **G** VASH1 expression levels in ovarian cancer and normal tissues. **H** Expression level of VASH1 mRNA in A2780 cells with or without MEG3 knockdown. **I** Expression level of VASH1 mRNA in SKOV3 cells with or without MEG3 overexpression. **J** Expression level of VASH1 protein in A2780 cells with or without MEG3 knockdown. **K** Expression level of VASH1 protein in SKOV3 cells with or without MEG3 overexpression. *P < 0.05; ***P < 0.001; ****P < 0.0001

This finding has aroused our interest in how MEG3 exerts its biological effects. The literature indicated that MEG3 deletion enhanced angiogenesis in vivo (provided by RefSeq). After predicting angiogenesis-related mRNAs with a strong correlation with MEG3 through the LinkedOmics website, we found that VASH1 had the strongest positive correlation with MEG3 (Spearman correlation = 0.31, P = 5.2e−11) (Fig. 5A–F), and the expression level of VASH1 decreased in ovarian cancer compared to normal tissue (Fig. 5G). To further verify the accuracy of the prediction results, we detected the expression of VASH1 in MEG3 knockdown and overexpression cell lines. The experimental results showed that after silencing MEG3, the mRNA and protein expression levels of VASH1 diminished in A2780 cells. Compared with the control group, the MEG3 overexpression group showed higher VASH1 mRNA and protein expression levels in SKOV3 cells (Fig. 5H–K).

### VASH1 suppressed the malignant phenotype of ovarian cancer cells

**Fig. 6:**
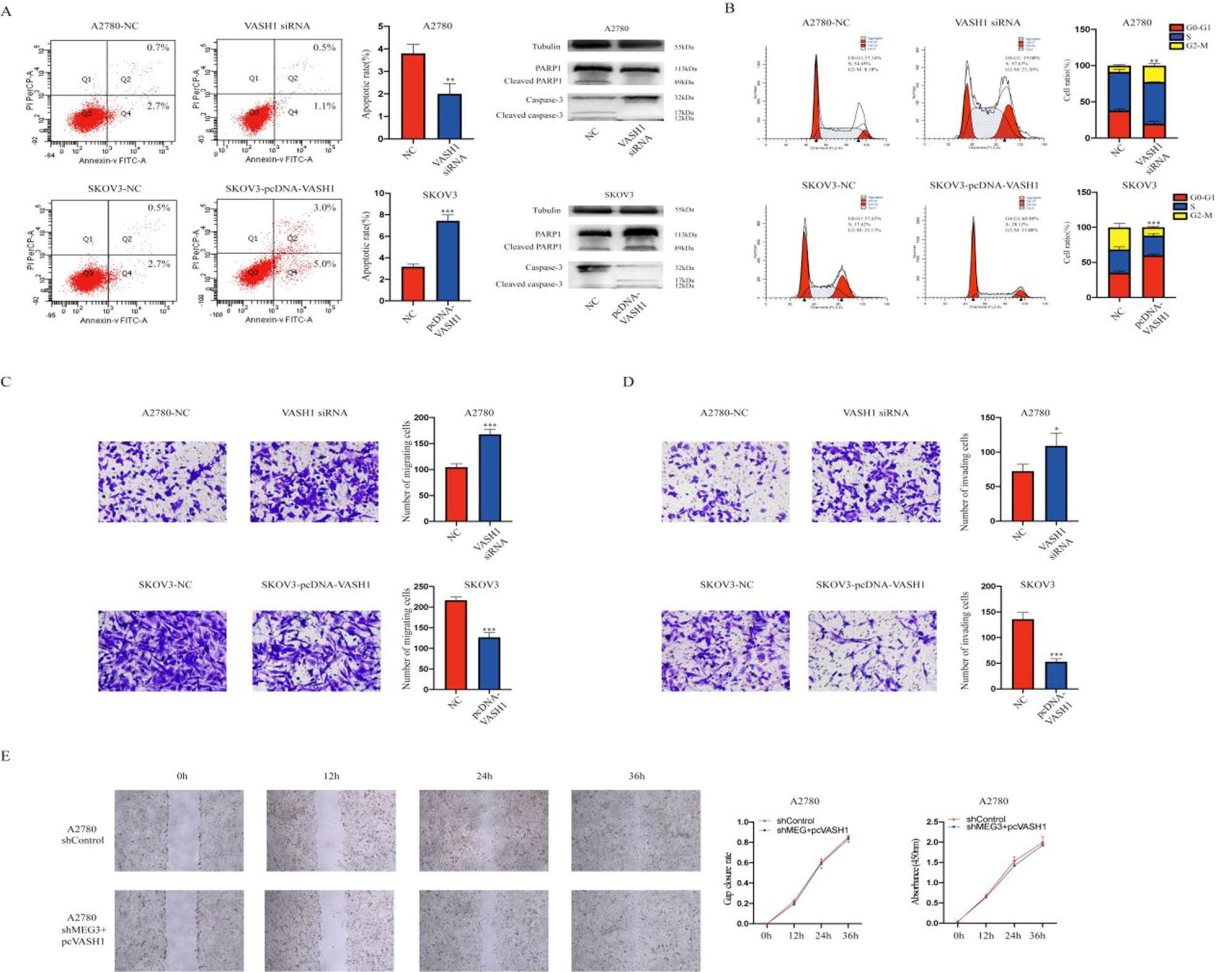
VASH1 can inhibit the malignant phenotype of ovarian cancer cells. **A** Apoptosis analysis of NC, VASH1-knockdown, and VASH1-overexpressing cells. **B** Cell cycle analysis of NC, VASH1 knockdown, and VASH1 overexpression cells. **C** Migration assay of NC, VASH1 knockdown, and VASH1 overexpression cells. **D** Invasion assay of NC, VASH1 knockdown, and VASH1 overexpression cells. **E** Scratch assay of NC and MEG3 knockdown + pc-VASH1 cells. *P < 0.05; **P < 0.01; ***P < 0.001

Our previous studies confirmed that VASH1 inhibited cell proliferation and wound healing ability [32]. Compared to the NC group of A2780 cells, the VASH1 siRNA group showed inhibited apoptosis and increased proliferation. Moreover, SKOV3 cells transfected with pcDNA-VASH1 presented a higher apoptosis rate and inhibition of the cell cycle than the NC group (Fig. 6A, B). Moreover, the VASH1 siRNA group was found to have increased migration and invasion abilities compared to the NC group, while the pcDNA-VASH1 group showed the opposite trends (Fig. 6C, D). After transfection of the VASH1 plasmid in MEG3 knockdown cell lines, there was no statistically significant difference in cell proliferation and migration abilities compared to those in the control group (Fig. 6E, F).

### MEG3 targets hsa-miR-885-5p and thereby regulates VASH1

**Fig. 7:**
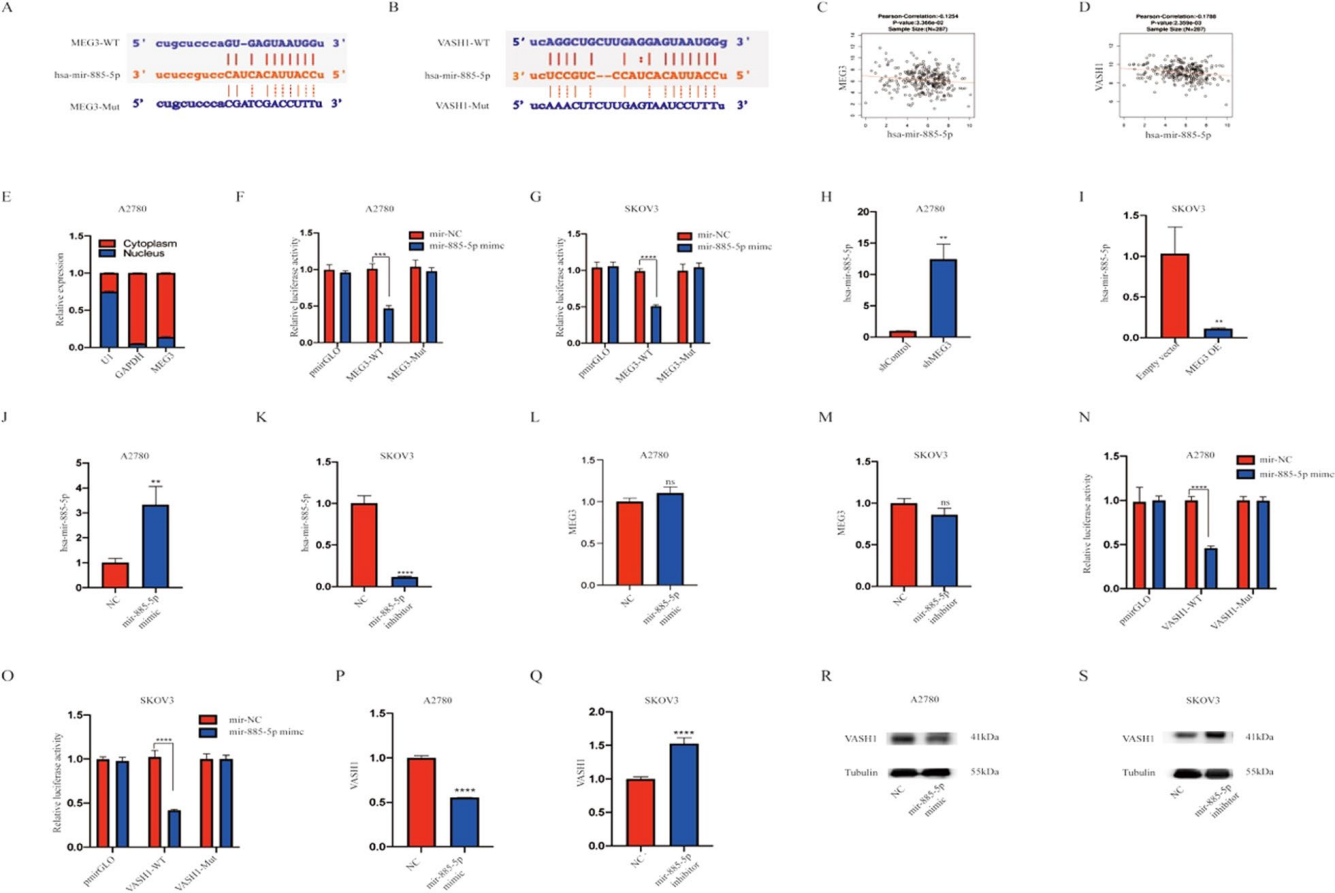
The regulatory relationship between MEG3, hsa-miR-885-5p, and VASH1. **A** The relationship between MEG3 and hsa-miR-885-5p. MEG3 sponges hsa-miR-885-5p at certain sites. **B** The correlation between hsa-miR-885-5p and VASH1. Hsa-miR-885-5p targets VASH1 at certain sites. **C** MEG3 expression is negatively correlated with the expression of hsa-miR-885-5p. **D** Hsa-miR-885-5p expression is negatively correlated with the expression of VASH1. **E** MEG3 is mainly expressed in the cytoplasm. **F**, **G** The luciferase activity of the hsa-miR-885-5p mimic + MEG3-Wt and hsa-miR-885-5p mimic + MEG3-Mut groups was compared with that of miR-NC group in A2780 and SKOV3 cell lines. **H**, **I** The impacts of MEG3 expression on hsa-miR-885-5p in A2780 and SKOV3 cell lines. **J**, **K** qRT‒PCR validation of the effects of the hsa-miR-885-5p mimic and inhibitor on the expression of hsa-miR-885-5p. **L**, **M** Hsa-miR-885-5p hardly affected MEG3 expression. **N**, **O** The luciferase activity of the hsa-miR-885-5p mimic + VASH1-Wt and hsa-miR-885-5p mimic + MEG3-Mut groups was compared with that of the miR-NC group in A2780 and SKOV3 cell lines. **P**, **Q** The impacts of hsa-miR-885-5p mimic and inhibitor treatment on VASH1 expression were examined by qRT‒PCR and **R**, **S** western blotting in A2780 and SKOV3 cell lines. **P < 0.01; ***P < 0.001; ****P < 0.0001

We predicted the microRNAs that form ceRNA regulatory relationships with MEG3 and VASH1 through the ENCORI website, and we ultimately selected hsa-mir-885-5p after screening out the previously reported microRNAs. We used starBase v2.0 software to predict the binding sites of MEG3 and hsa-miR-885-5p (Fig. 7A). The LinkedOmics website was used to predict the correlation between hsa-miR-885-5p, MEG3, and VASH1 (Fig. 7B). After separating the total RNA from the cytoplasm and nucleus, we confirmed that MEG3 was mainly expressed in the cytoplasm (Fig. 7C, D). Subsequently, the dual-luciferase reporter assay showed that compared to cells transfected with miR-NC and MEG3-WT, cells transfected with hsa-miR-885-5p mimic and MEG3-WT exhibited lower luciferase activity. However, there was no difference in luciferase activity between the hsa-miR-885-5p mimic + MEG3-Mut and miR-NC + MEG3-Mut groups (Fig. 7E, F). In addition, we found that knocking down the expression of MEG3 contributed to upregulation of hsa-miR-885-5p, while overexpression of MEG3 downregulated hsa-miR-885-5p (Fig. 7H, I). However, variation in hsa-miR-885-5p expression led to minimal change in MEG3 expression (Fig. 7J–M). The luciferase activity of the hsa-miR-885-5p mimic + VASH1-Wt group was significantly lower than that of the miR-NC + VASH1-Wt group, but there was almost no difference detected between the hsa-miR-885-5p mimic + VASH1-Mut and miR-NC + VASH1-Mut groups (Fig. 7N, O). In addition, transfection with the hsa-miR-885-5p mimic significantly reduced the expression levels of VASH1 mRNA and protein. Transfection of the cells with the hsa-miR-885-5p inhibitor led to an increase in VASH1 expression (Fig. 7P–S).

### MEG3 inhibited tumor growth and regulated VASH1 in vivo

**Fig. 8:**
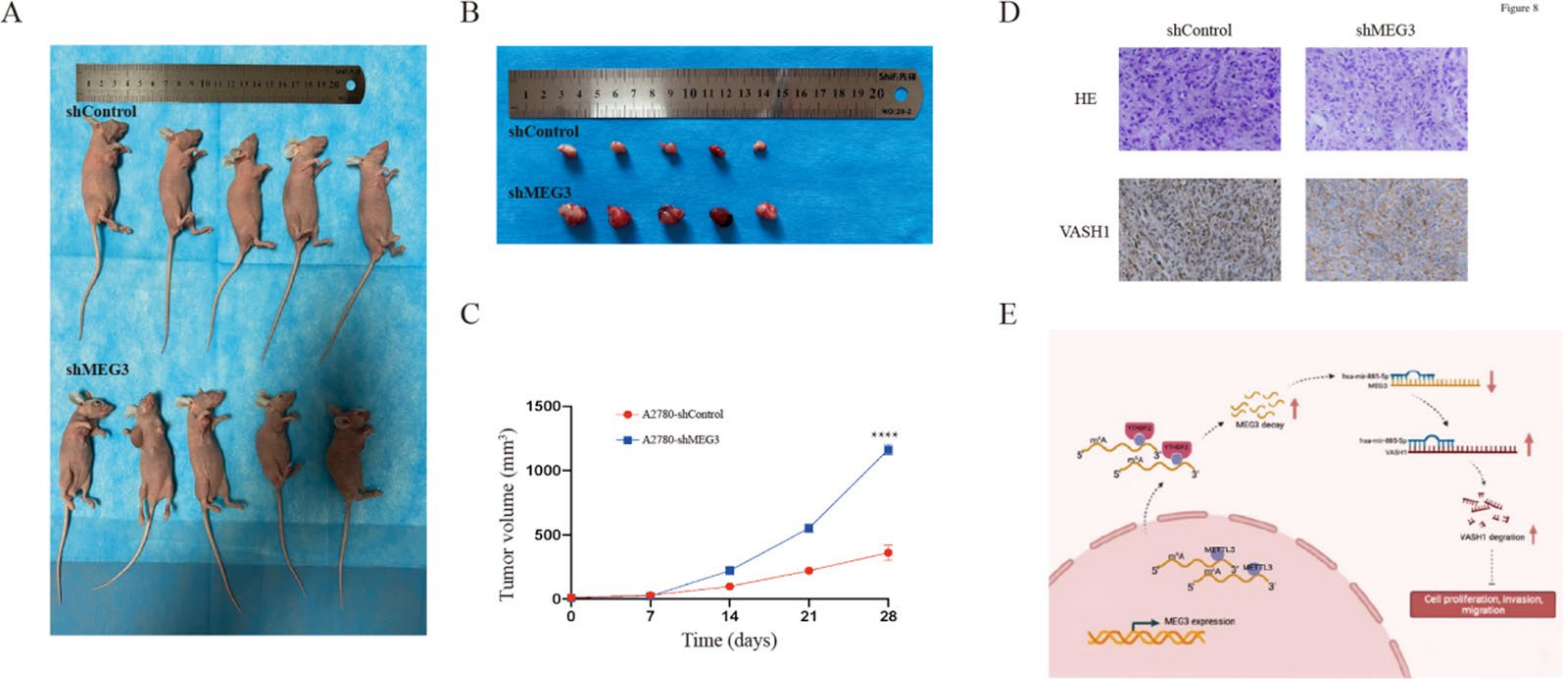
MEG3 inhibited tumor growth in an orthotopic xenograft model. **A**, **B** Mice and tumor images at 28 days after tumor implantation in an orthotopic xenograft model generated by A2780 cells transfected with an empty vector and shMEG3. **C** Tumor volume growth curve. **D** Representative images of H&E and immunohistochemical staining for VASH1 in the tumors of nude mice. **E** The mechanistic scheme: METTL3-induced YTHDF2 m^6^A methylation regulates the MEG3/hsa-miR-885-5p/VASH1 axis and suppresses ovarian cancer malignancy

To confirm whether MEG3 affects tumor growth in vivo, A2780 cells transfected with a scramble vector or shMEG3 were injected into nude mice. After 4 weeks of continuous monitoring, we found that the tumors in the shMEG3 cohort were remarkably larger than those in the scramble cohort (Fig. 8A, B). Moreover, the average tumor volumes were markedly higher in the shMEG3 cohort than in the scramble cohort (Fig. 8C). Finally, VASH1 was detected by IHC staining to analyze its correlation with MEG3 expression. The results indicated that the VASH1 level was decreased in shMEG3 xenografts (Fig. 8D). These results support a role for MEG3 in suppressing ovarian cancer cell growth in vivo.

## Discussion

The pathogenesis of ovarian cancer is complex, with nonspecific symptoms, making treatment difficult for patients with advanced ovarian cancer. Therefore, seeking suitable biomarkers for the diagnosis of early ovarian cancer is an urgent issue that needs to be addressed, and identifying effective molecular targets for the treatment of ovarian cancer should also be given priority consideration.

In recent years, with the development of second-generation sequencing technology, research has shown that lncRNAs play an important role in many biological fields, such as tumor development and oncogenesis, and are important regulatory molecules in the human genome. LncRNAs can regulate gene expression at epigenetic, transcriptional, or posttranscriptional levels by interacting with proteins, DNA, and RNA and participate in important processes such as chromatin nuclear transport, regulation of oncogene activation, immune cell differentiation, and immune system regulation [33]. LncRNAs have mostly been studied in depth in the context of cancer. The occurrence and development of cancer can be mediated by various mechanisms of lncRNAs, mainly through epigenetic regulation, activation of carcinogenic pathways, and interference from other RNA subtypes [34]. In our study, we first screened lncRNAs with decreased expression in gynecological tumors through bioinformatics analysis and then sorted them based on the degree of expression difference. At this time, MEG3, which ranked first, attracted our interest. Previous studies have confirmed that MEG3 plays an antitumor role in various other cancers, including gynecological malignancies. For example, in prostate cancer, MEG3 inhibits progression by regulating the miR-9-5p/QKI-5 axis [35]. Similarly, MEG3 inhibits laryngeal cancer cell proliferation and induces cell apoptosis by regulating APAF-1 [36]. The anticancer effect of MEG3 also occurs in tongue squamous cell carcinoma [37], glioma [38], neuroblastoma [39], cervical cancer [23], endometrial cancer [40], ovarian cancer [41] and others. Consistent with the above research, our study shows that compared to that in normal tissues, MEG3 expression is generally reduced in pancancer tissues, and the difference is most significant in ovarian cancer. Upregulation of MEG3 expression is associated with a better prognosis in ovarian cancer patients (Fig. 1). In addition, silencing MEG3 results in faster proliferation, stronger migration, and delayed apoptosis of ovarian cancer cells (Figs. 2, 3).

With the deepening of research, there are an increasing number of mechanisms related to lncRNAs. Current research suggests that m^6^A modification can serve as a structural “switch” to alter the conformation of lncRNAs, participate in the ceRNA model of silencing miRNAs, or affect the stability and expression of lncRNAs [42]. Moreover, m^6^A modification promotes the absorption of downstream miRNAs as endogenous RNA by enhancing the stability of lncRNA transcripts, thereby reducing mRNA degradation [43]. To identify proteins that regulate MEG3 m^6^A methylation among numerous m^6^A “writers” and “readers”, we predicted methylation-related proteins that can bind to MEG3 through the database. The results suggested that METTL3 and YTHDF2 potentially regulate MEG3 methylation. In recent years, accumulated evidence has shown that METTL3 plays a crucial role in various types of cancer, depending on or independent of its m^6^A RNA methyltransferase activity. In most cases, METTL3 has been reported as an oncogenic gene that promotes the occurrence and development of various cancers, including hematopoietic malignancies and solid tumors, by depositing m^6^A modifications on key transcripts [44–47]. In ovarian cancer cells, METTL3 served as an oncogenic gene promoting tumor growth and invasion (Additional file 3). YTHDF2 belongs to the YT521-B homology (YTH) domain family, and its function is to partially promote the degradation of target transcripts by recruiting CCR4-NOT enzyme complexes [48]. YTHDF2-mediated m^6^A modification is critical for posttranscriptional regulation of target genes [49]. Previous studies have shown that METTL3 mediates the m^6^A modification of SOCS2 and promotes its mRNA degradation through a YTHDF2-dependent pathway [50]. YTHDF2 mediates the mRNA degradation of the tumor suppressors LHPP and NKX3-1 in a METTL3-dependent manner to regulate AKT phosphorylation-induced prostate cancer progression [51]. Similarly, YTHDF2 mainly acted as an m6A “reader” to degrade mRNA in ovarian cancer cells [52–54]. Meanwhile, studies have shown that YTHDF2 is significantly upregulated in ovarian cancer tissue. Further functional experiments have confirmed that YTHDF2 significantly promotes the proliferation, migration, and invasion of OC cell lines (Additional file 4). Our research results confirmed that YTHDF2 accelerated MEG3 degradation mediated by METTL3 methylation, indicating that it is a negative regulatory factor (Fig. 4).

In addition, MEG3 has been confirmed to target hsa-miR-885-5p, thereby upregulating the expression of VASH1 and exerting an inhibitory effect on ovarian cancer (Fig. 5). To date, there has been very little research on hsa-mir-885-5p in tumors. We only know that hsa-miR-885-5p, as a carcinogenic factor, is associated with the poor prognosis of breast cancer patients [55]. We also found that VASH1 leads to an increase in the proportion of apoptosis and a decrease in ovarian cancer cell proliferation, invasion, and migration ability (Fig. 6). The human VASH1 gene is located on chromosome 14q24.3, with a protein molecular weight of 44 kD [56]. VASH1 is a member of the angiostatin protein family. As an endogenous angiogenesis inhibitor, VASH1 is induced by vascular endothelial growth factor (VEGF) and fibroblast growth Factor 2 (FGF2), which inhibits angiogenesis through a negative feedback mechanism under physiological conditions [57]. Research has shown that VASH1 plays a major role in various malignant tumors, including prostate cancer, upper urinary tract epithelial cancer, cancer lung cancer, and cervical tumors [57–61]. As a key angiogenic regulator, VASH1 inhibits tumor angiogenesis and growth in animal tumor models of lung cancer [62] and hepatocellular carcinoma [63]. In addition, low VASH1 levels are correlated with large tumor size, advanced clinical staging, and distant metastasis in colon cancer patients [64]. In our research, hsa-miR-885-5p was found to be involved in the regulation of VASH1 as a competitive RNA for MEG3 and VASH1. MEG3 positively regulated the expression level of VASH1 in ovarian cancer cells through hsa-miR-885-5p, exerting an antitumor effect (Fig. 7).

In this study, we comprehensively demonstrated that lncRNA MEG3 is regulated by METTL3-mediated YTHDF2 methylation; it upregulates VASH1 through regulatory competitive binding to hsa-miR-885-5p and then inhibits ovarian cancer cell proliferation, migration, and invasion. In many types of cancer, dysregulation of MEG3 has been reported, and its tumor inhibitory activity is mediated through interactions with p53-dependent transcription or Rb-related pathways [65–68]. The novelty of this study lies in revealing a new mechanism of MEG3 in human ovarian cancer cells. New strategies based on lncRNAs are expected to be developed to treat ovarian cancer. However, the content of this study is limited by the lack of clinical patient samples, so we will expand the sample size in the future.

## Conclusions

In summary, our research results indicate that MEG3 expression is reduced in ovarian cancer.

MEG3 knockdown facilitated the malignancy of ovarian cancer in both in vivo and in vivo models. Mechanistically, YTHDF2 accelerates the degradation of MEG3 by recognizing the m^6^A modification mediated by METTL3. MEG3 enhances the expression of VASH1 via competitive binding to hsa-miR-885-5p. Overall, our study confirms the inhibitory effect of MEG3 in ovarian cancer and reveals new regulatory mechanisms of MEG3 from the perspectives of m^6^A methylation and ceRNA, and our results are expected to provide new targets for the treatment of ovarian cancer.

### Supplementary Information

**Additional file 1: **The name of the top 50 downregulated genes in gynecological malignancies, including ovarian cancer, cervical cancer, endometrial cancer, and uterine sarcoma.

**Additional file 2: **MEG3 could inhibit the proliferation of ovarian cancer cells. **A** qRT‒PCR was used to verify the construction of MEG3 knockdown in SKOV3 cell lines. **B** CCK-8 assay of NC and MEG3 knockdown cells. **C** Colony formation assay of NC and MEG3 knockdown cells. **D** Cell cycle analysis of NC and MEG3 knockdown cells. **E** Migration assay of NC and MEG3 knockdown cells. **F** Invasion assay of NC and MEG3 knockdown cells. *P < 0.05; **P < 0.01; ***P < 0.001; ****P < 0.0001.

**Additional file 3: **METTL3 could facilitate the malignant phenotype of ovarian cancer cells. **A** Cell cycle analysis of cells with or without METTL3 overexpression. **B** Cell cycle analysis of cells with or without METTL3 siRNA. **C** Transwell analysis of cells with or without METTL3 overexpression. **D** Transwell analysis of cells with or without METTL3 siRNA. **E** Gap closure rate of cells with or without METTL3 overexpression, METTL3 siRNA. **F** CCK8 analysis of cells with or without METTL3 overexpression, METTL3 siRNA.

**Additional file 4: **YTHDF2 could facilitate the malignant phenotype of ovarian cancer cells. **A** Cell cycle analysis of cells with or without YTHDF2 overexpression. **B** Cell cycle analysis of cells with or without YTHDF2 siRNA. **C** Transwell analysis of cells with or without YTHDF2 overexpression. **D** Transwell analysis of cells with or without YTHDF2 siRNA. **E** Gap closure rate of cells with or without YTHDF2 overexpression, YTHDF2 siRNA. **F** CCK8 analysis of cells with or without YTHDF2 overexpression, YTHDF2 siRNA.

## Data Availability

The datasets analysed during the current study are available in the TCGA.
