# Supplementary figures and images for: m6A methylation-mediated regulation of LncRNA MEG3 suppresses ovarian cancer progression through miR-885-5p and the VASH1 pathway
Source: J Transl Med. 2024 Jan 29;22:113. doi: 10.1186/s12967-024-04929-x (PMC10823642; doi:10.1186/s12967-024-04929-x)

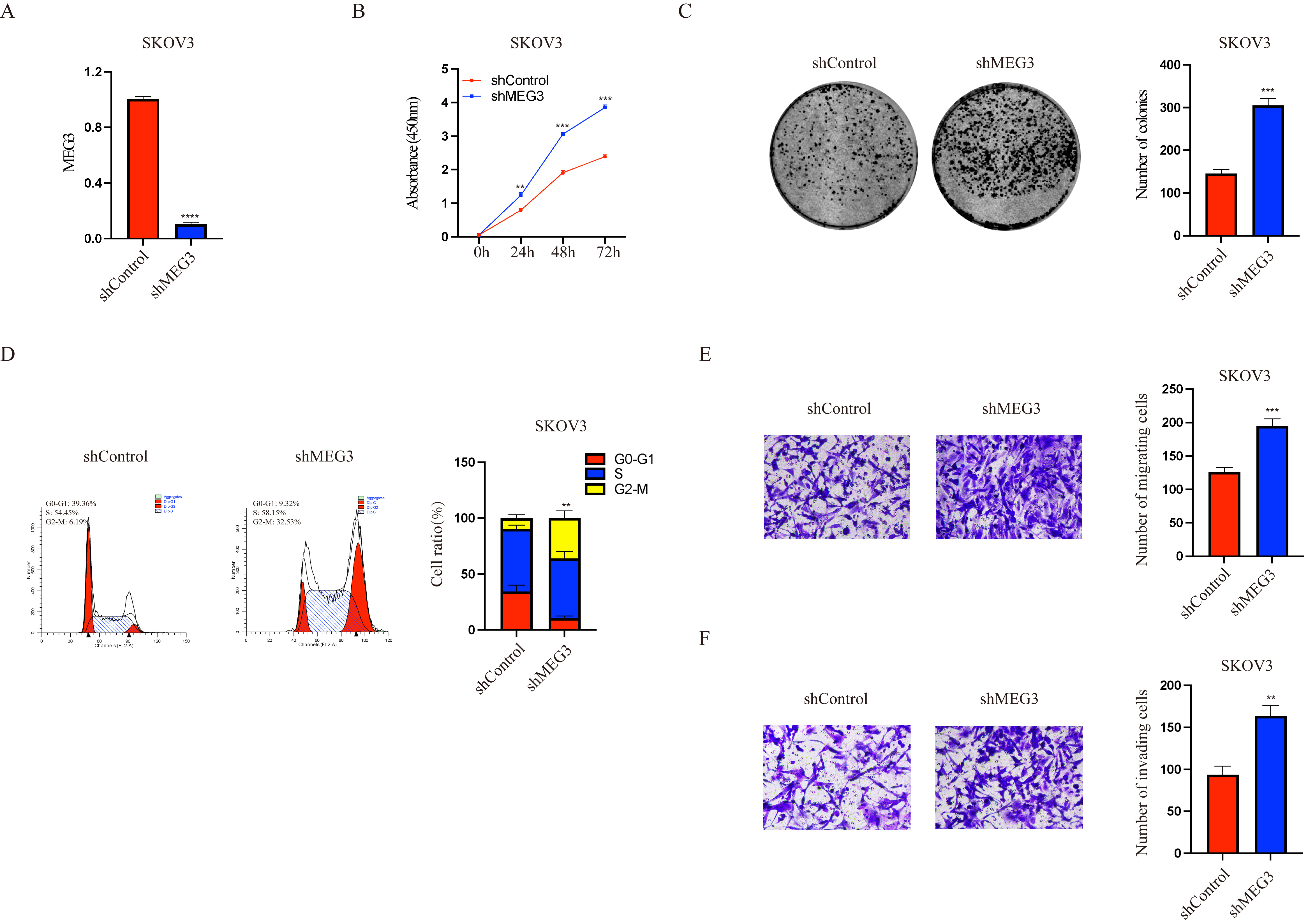

Supplement: Supplementary file 2 — Additional file 2: MEG3 could inhibit the proliferation of ovarian cancer cells. A qRT‒PCR was used to verify the construction of MEG3 knockdown in SKOV3 cell lines. B CCK-8 assay of NC and MEG3 knockdown cells. C Colony formation assay of NC and MEG3 knockdown cells. D Cell cycle analysis of NC and MEG3 knockdown cells. E Migration assay of NC and MEG3 knockdown cells. F Invasion assay of NC and MEG3 knockdown cells. *P < 0.05; **P < 0.01; ***P < 0.001; ****P < 0.0001. [file 12967_2024_4929_MOESM2_ESM.jpg]

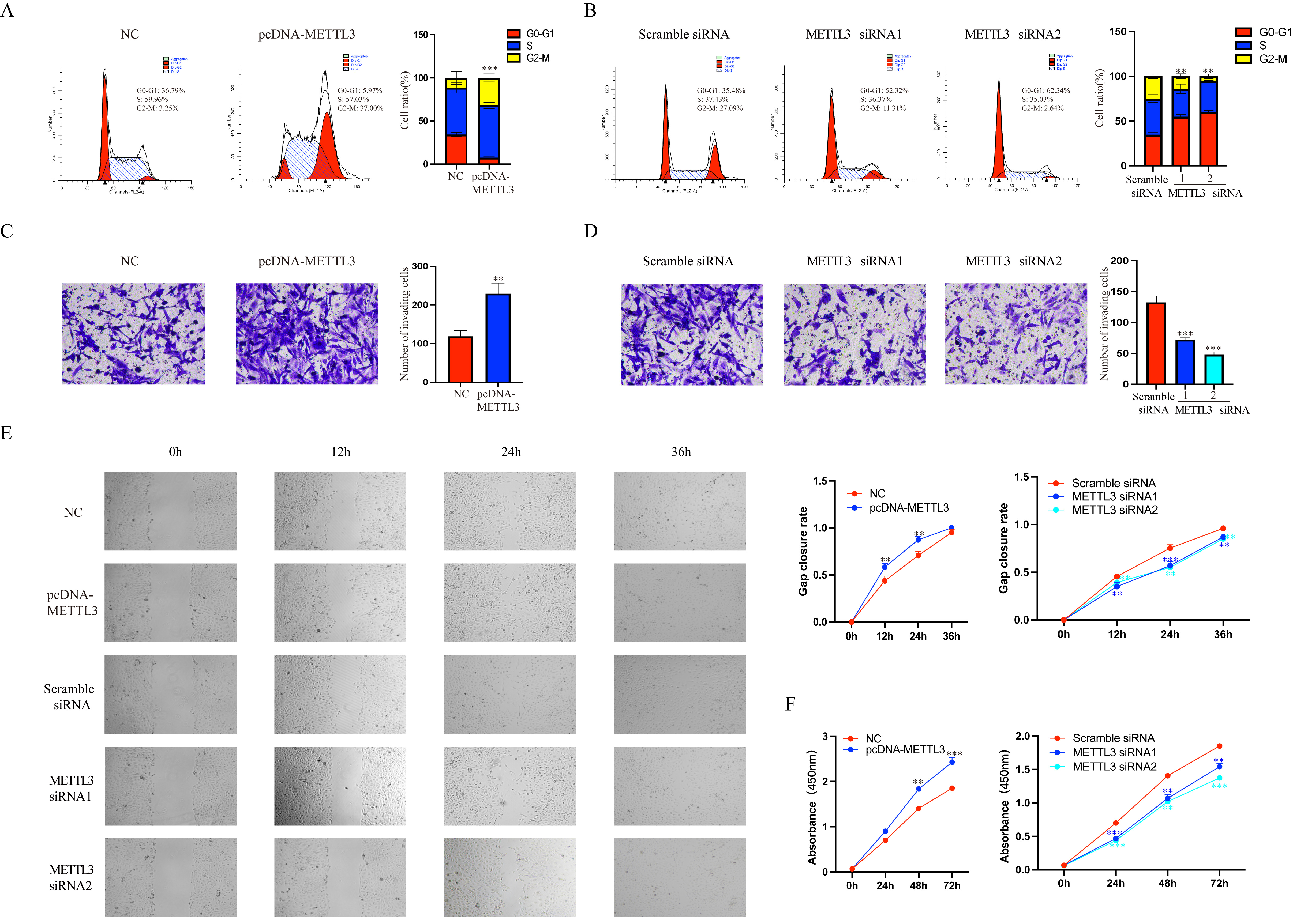

Supplement: Supplementary file 3 — Additional file 3: METTL3 could facilitate the malignant phenotype of ovarian cancer cells. A Cell cycle analysis of cells with or without METTL3 overexpression. B Cell cycle analysis of cells with or without METTL3 siRNA. C Transwell analysis of cells with or without METTL3 overexpression. D Transwell analysis of cells with or without METTL3 siRNA. E Gap closure rate of cells with or without METTL3 overexpression, METTL3 siRNA. F CCK8 analysis of cells with or without METTL3 overexpression, METTL3 siRNA. [file 12967_2024_4929_MOESM3_ESM.jpg]

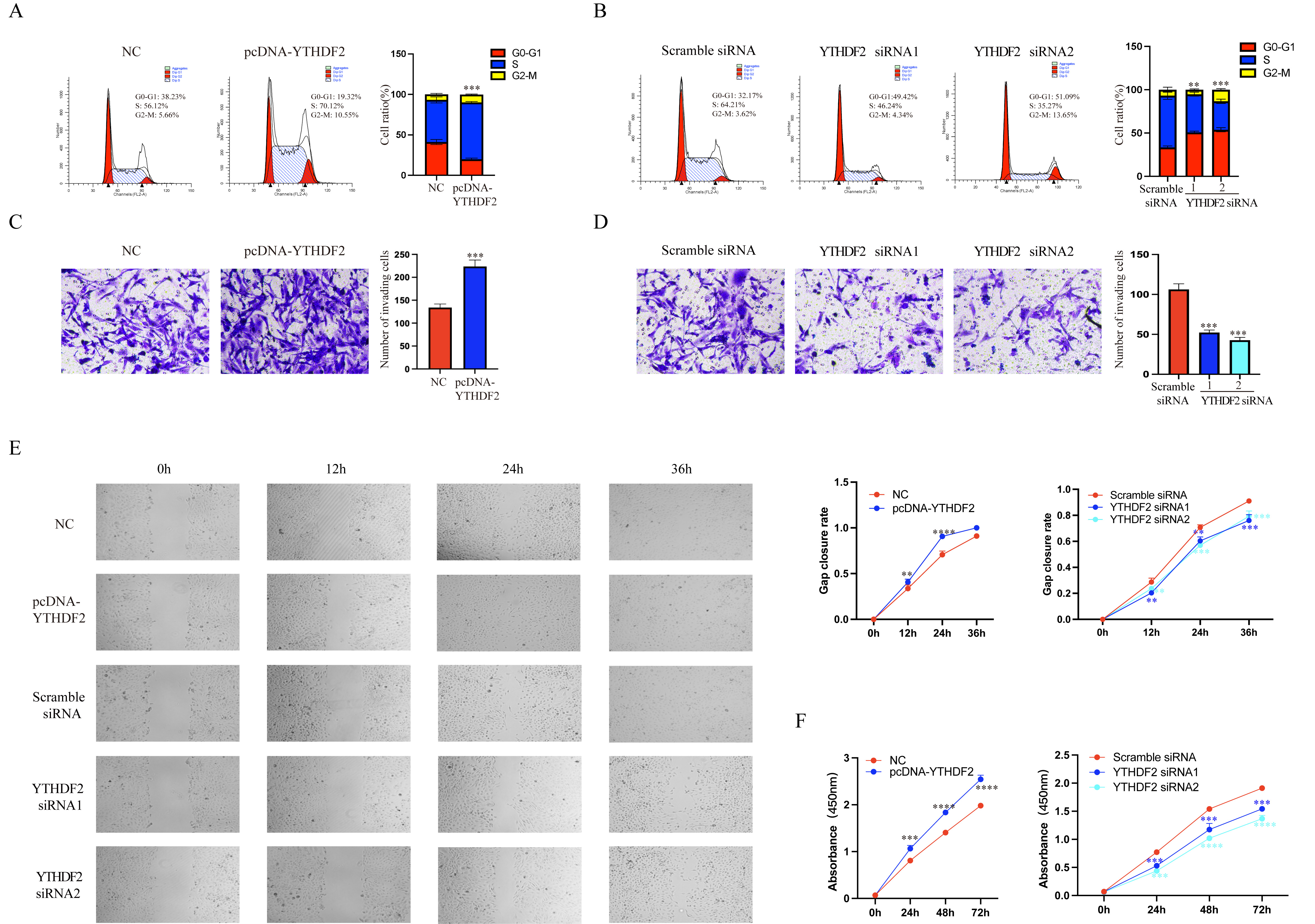

Supplement: Supplementary file 4 — Additional file 4: YTHDF2 could facilitate the malignant phenotype of ovarian cancer cells. A Cell cycle analysis of cells with or without YTHDF2 overexpression. B Cell cycle analysis of cells with or without YTHDF2 siRNA. C Transwell analysis of cells with or without YTHDF2 overexpression. D Transwell analysis of cells with or without YTHDF2 siRNA. E Gap closure rate of cells with or without YTHDF2 overexpression, YTHDF2 siRNA. F CCK8 analysis of cells with or without YTHDF2 overexpression, YTHDF2 siRNA. [file 12967_2024_4929_MOESM4_ESM.jpg]
